# Small bowel adenocarcinoma - *terra incognita*: A demand for cross-national pooling of data

**DOI:** 10.3892/ol.2014.1919

**Published:** 2014-02-27

**Authors:** KATRIN SCHWAMEIS, SEBASTIAN FRIEDRICH SCHOPPMANN, JUDITH STIFT, MICHAEL SCHWAMEIS, ANTON STIFT

**Affiliations:** 1 Gastroesophageal Tumor Unit, Comprehensive Cancer Center, Vienna A-1090, Austria; 2 Department of Surgery, Medical University of Vienna, Vienna A-1090, Austria; 3 Department of Pathology, Medical University of Vienna, Vienna A-1090, Austria; 4 Department of Clinical Pharmacology, Medical University of Vienna, Vienna A-1090, Austria

**Keywords:** small bowel adenocarcinoma, data pooling, multinational, surgery, chemotherapy

## Abstract

To date, due to the rarity, tumor biology and carcinogenesis of small bowel adenocarcinoma (SBA), the disease has been explored insufficiently and immunophenotyping and molecular characterization have not been finalized. This knowledge gap consecutively leads to an overt lack of diagnostic and therapeutic recommendations. In the current study, we provide our experience with the treatment of SBA, and demand for cross-national data pooling to enable unlimited information transfer and higher powered study. A comprehensive database of all patients with SBA was established and consecutively reviewed for clinicopathohistological data, information concerning preoperative evaluation, surgical and chemotherapeutical treatment, as well as outcome parameters. Patients underwent curative intended surgery (42.4%; n=14), adjuvant chemotherapy (CTX) following resection (36.4%; n=12) or palliative care (21.2%; n=7). The majority of patients were diagnosed at an advanced disease stage (pT3, 36.4%; pT4, 39.4%) and the duodenum was the most common tumor site (57.1%; n=20). Complete surgical resection was achieved in 88.5% of patients, while postoperative complications occurred in 19.4%. Within a mean follow-up period of 31.4 months, 17 patients succumbed to the disease following a median survival time of 11 months. Mean overall survival (OS) was 47.4, 25.3 and 9.8 months for surgically, surgically and chemotherapeutically and palliatively treated patients, respectively. Early surgical resection remains the mainstay in the treatment of localized SBA, since it is associated with a prolongation of OS. The role of neoadjuvant and adjuvant CTX has not yet been defined. Thus, since no consensus exists on the adequate treatment of these malignancies, we demand an international collaboration and cross-national data pooling to pave the way for the implementation of evidence-based standard care operating procedures.

## Introduction

Despite an increasing incidence, particularly in duodenal adenocarcinoma, small intestine malignancies are rare, accounting for ~2% of all gastrointestinal tumors. Adenocarcinoma is the most common histopathological subtype (1), followed by carcinoid tumors, lymphomas and sarcomas (2,3).

In accordance with the Robert Koch Institute in Germany, ~0.33 per 10^5^ males and 0.24 per 10^5^ females are diagnosed with primary adenocarcinoma of the small intestine each year. The incidence rates of small bowel adenocarcinoma (SBA) in the U.S. population are 1.45 and 1.00 for males and females per 10^5^ individuals each year, respectively (4).

SBA is 40–50 times less common than colorectal carcinoma, although the small intestine accounts for 70–80% of the total length and ~90% of the overall surface of the gastrointestinal tract (5). The reason for this distinct difference in incidence remains unclear. In total, ~50% of all SBA are located in the duodenum, most commonly in the second portion near to the papilla of Vater, 30% arise in the jejunum and the remaining fifth occur within the ileum. Diagnosis is mainly determined in middle-aged to elderly patients (in their fifth and sixth decades of life) with higher prevalence rates in individuals of African descent than in Caucasians (1).

The most important predisposing condition recognized for SBA is Crohn’s disease, followed by celiac disease, Meckel’s diverticulum, intestinal duplication and hereditary cancer syndromes [i.e. hereditary intestinal polyposis syndrome (Peutz-Jeghers syndrome), familial adenomatous polyposis, hereditary non-polyposis colon cancer syndrome and familial colorectal polyposis (Gardner syndrome)]. Known independent prognostic factors indicating poor outcome are lymph node metastasis ratio and distal tumor location (i.e. jejunum and ileum) (3,6,7).

Following a review of 491 cases of SBA, the Mayo Clinic reported that higher age, male gender, increased TNM stage and grade, residual disease following resection and a lymph node ratio of ≥50% predict decreased overall survival (OS) in univariate analysis, with age and TNM staging being predictive for survival in multivariate analysis (8). Previously, Overman *et al* demonstrated a distinctly poorer cancer-specific survival in SBA than in large bowel adenocarcinoma (9).

The ongoing poor prognosis of SBA with an overall five-year survival rate of ~25%, even following complete surgical (R0) resection and adequate lymphadenectomy, is mainly attributable to vague, non-specific symptoms, varying accessibility to endoscopy and the lack of evidence-based diagnostic procedures resulting in long latency time to diagnosis (10). Thus, despite increasing advantages in radiographic imaging, early detection of small bowel neoplasms remains infrequent and the majority of patients present with already unresectable or metastatic disease (1). Due to the rarity of these tumors, there is an ongoing lack of sufficient data characterizing this patient population adequately.

The molecular characterization of colorectal cancer has led to a differentiated understanding of tumorigenesis and has resulted in a revolution and individualization of therapy options. Recent literature provides little data on the etiopathogenesis, tumor biology and molecular pathways of SBA. A more sophisticated understanding of carcinogenesis is essential for further hypothesis generation and the development of new individualized and targeted therapeutic approaches. To establish treatment guidelines and define predictors of prognosis, translational study on SBA is highly warranted. Surgery is the mainstay of therapy. Concerning adjuvant chemotherapy (CTX), only a few recommendations with higher levels of evidence are available and its role for resectable carcinoma remains unclear (5). Thus, data on first line CTX regimen in advanced stages are also scarce.

The current study presents a consecutively collected case series of 33 SBA patients. The aim of the study was to share our experience of SBA treatment as a high-volume center, and to provide a potential basis for multinational data pooling and cross-national research collaboration.

## Materials and methods

### Patient characteristics

A database of all patients with histologically verified malignancy of the small intestine, who were diagnosed at the Department of Surgery, Medical University of Vienna (Vienna, Austria), between 1994 and 2012, was established. All tumors others than primary adenocarcinoma of the small intestine were excluded. Since primary adenocarcinoma of the major duodenal papilla represent a separate tumor entity, those tumors were also excluded. This led to an inclusion of 33 patients, who were reviewed for demographic data (age, gender and comorbidities), baseline characteristics (clinical manifestation and primary complaints), predisposing conditions and prognostic factors, tumor features, preoperative diagnostics, surgical and medical treatment patterns, and outcome parameters. The study protocol was approved by the ethics committee of the Medical University of Vienna (no. EC 242/2009).

### Pathohistological analysis

Final SBA diagnosis was determined by pathohistological analysis performed at the Clinical Institute of Pathology, Medical University of Vienna.

### Statistical analysis

Statistical calculations were performed using IBM SPSS^®^ statistics 19.0 (SPSS, Inc., Chicago, IL, USA). Data are presented as the means ± SD or the median and interquartile range (IQR), respectively. OS rates were calculated using the Kaplan-Meier method and defined as the time from surgical resection to mortality or last follow-up visit. A two-sided P-value of <0.05 was considered to indicate a statistically significant difference.

## Results

### Patient analysis

A total of 33 patients (20 males and 13 females) were diagnosed with primary SBA at a median age of 63 years (IQR, 24–83 years) between 1994 and 2012 at the Department of Surgery, Medical University of Vienna. The most common tumor sites were the duodenum (n=20; 60.6%) and jejunum (n=8; 24.2%) (Table I). Frequent observations at initial admission were sporadic abdominal discomfort (n=21; 63.6%), including abdominal pain and fullness and meteorism, as well as hypo- or normochromatic anemia (mean hemoglobin level, 10.4 mg/dl) due to occult blood loss (n=15; 45.5%). Overall weight loss was documented in 14 cases (42.4%; mean loss, 11.5 kg over 3 weeks). In total, four patients presented with clinical signs of gastric orifice stenosis and three patients were admitted to our institution complaining of vomiting, nausea and abdominal pain caused by mechanical bowel obstruction. One patient suffered from chest pain and dyspnea due to thromboembolic disease, likely caused by underlying malignant disease.

### Patient diagnosis

Initial diagnosis was determined mainly by high-resolution computed tomography of the abdomen, followed by or based on an esophagogastroduodenoscopy procedure.

SBA predisposing conditions were found in three cases, consisting of Morbus Crohn, celiac disease and familial adenomatous polyposis. With regard to metachronous malignant neoplasms, one gastric cancer and two types of colorectal cancer were identified.

Elevated β2-microglobulin levels were found in nine of the 12 patients (mean, 2.0 mg/l; reference range, 0–1.9 mg/l), pathological CA 19-9 values were measured in seven of the 24 patients (mean, 506.4 kU/l; reference range, 0–37 kU/l) and an increased CEA level was observed in only five out of 25 patients (mean, 24.8 μg/l; reference range, 0–3.4 μg/l).

### Treatment and tumor classification

All patients, with the exception of two, underwent surgical treatment. In total, 26 patients were treated with primary curative intent, including adequate lymphadenectomy, of whom the majority received small bowel segmental resection (n=20; 60.0%) and six (18.2%) patients received partial pancreatoduodenectomy with Y-Roux anastomosis. In five cases (15.2%), a palliative gastroenterostomy was accomplished (Table II).

R0 resection was performed in 23 patients (88.5%), while in one case (3.8%), only incomplete resection was achieved. Regardless of surgical procedure, lymph node metastases were found in 14 patients (45.2%), while 13 patients (41.9%) were staged as pN0 (Table I).

According to the Union for International Cancer Control TNM classification (11), 75.8% of patients were staged as pT3 (36.4%) or pT4 (39.4%) and nine patients suffered from carcinosis peritonei (27.3%). Histopathologically, adenocarcinoma were classified into well-differentiated (G1; n=3; 9.1%), moderately differentiated (G2; n=18; 54.5%) and poorly differentiated (G3; n=12; 36.4%) groups.

Postoperative complications occurred in six patients (19.4%), including four anastomotic leaks (12.9%) and two wound infections (6.5%). According to the Clavien-Dindo classification, four patients were staged as IIIb, two patients as stage I and the remaining 25 surgically treated individuals were staged as 0.

CTX was performed in 17 cases (51.5%). The majority of patients were subjected to oxaliplatin and capecitabine (XELOX or CAPOX), fluorouracil, leucovorin and irinotecan (FOLFIRI) and folinic acid, fluorouracil and oxaliplatin (FOLFOX) regimens with no detectable differences in terms of survival.

One patient received gemcitabine neoadjuvantly, followed by performance of a gastroenterostomy with hepaticojejunostomy and adjuvant XELOX application.

### Follow-up

Within a mean follow-up period of 31.4 months and following a median survival time of 11 months, 17 patients (51.5%) succumbed to the disease, of whom five had received primary palliative surgical care (gastroenterostomy) while two patients had not undergone any surgical therapy.

**Figure 1:**
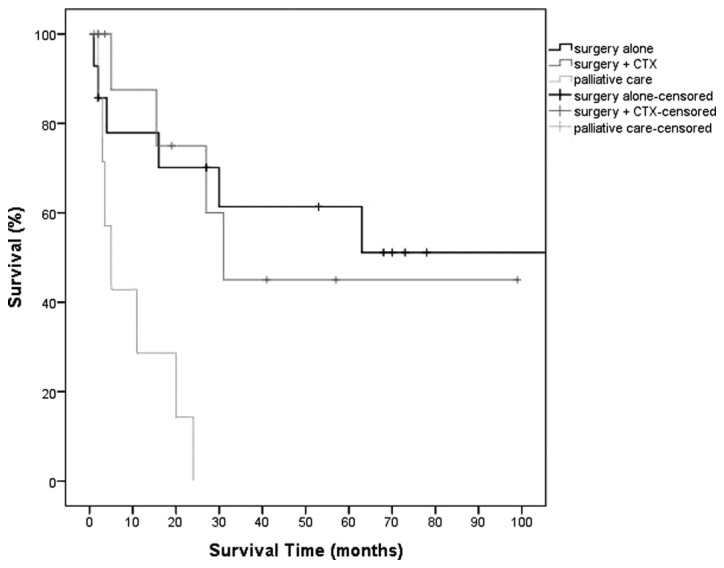
Survival curves with respect to performed therapy. CTX, chemotherapy.

**Figure 2:**
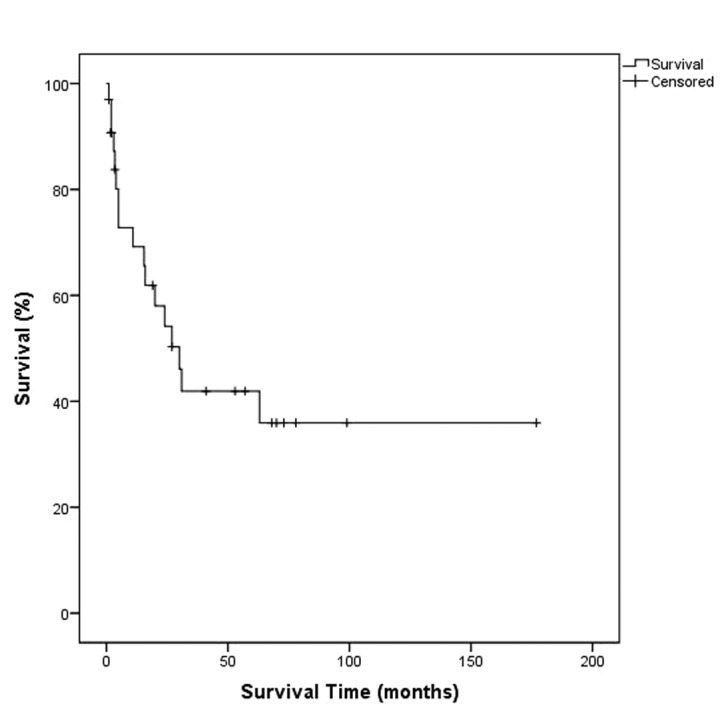
Overall survival.

Following selective surgery, the mean OS was 47.4 months and the mean survival time was 19.3 months, while the mean OS was 25.3 months and the mean survival time was 19.6 months for those who had received adjuvant CTX. The remaining patients, who all underwent palliative care and all succumbed to the disease within the follow-up period, exhibited a mean OS of 9.8 months (Table III; Figs. 1 and 2).

## Discussion

SBA carries a poor prognosis and diagnosis is usually determined extremely late at advanced stage. At present, no established screening methods or diagnosis protocols exist and SBA remains a diagnostic and therapeutic challenge with a markedly increasing incidence during the last 50 years (2). The non-specificity of initial clinical complaints and, in part, the endoscopic inaccessibility are major factors substantially contributing to the delayed diagnosis with an average latency time of ~8.2 months, resulting in a distribution of pT staging of ~90% pT3 or pT4 at initial diagnosis (3). The implementation of evidence-based procedures for detection at an early stage is urgently required to enhance resectability rates.

In the current series, patients mostly suffered from fatigue or dyspnea due to anemia or from non-specific abdominal discomfort. Accordingly, Poddar *et al* recently emphasized the importance of considering SBA as a possible underlying cause of unexplained iron deficiency anemia (1).

Early surgery represents the mainstay in therapy and the only opportunity for cure (10). There is an overt lack of evidence-based therapeutic recommendations, but no consensus exists on the adequate treatment of these malignancies. This is largely due to the rarity of SBA. Consecutively, studies performed to date, comprising only small sample sizes, are less conclusive.

Furthermore, the role of adjuvant CTX in curatively resectable SBA remains unclear and no standard first line CTX regimen for advanced disease has yet been established. According to the previous literature, only non-significant survival benefits have been shown for adjuvant CTX (7,8,10,12–17) and, to date, no prospective trials addressing this issue have been published. In 2010, a retrospective study performed by Overman *et al* demonstrated an improvement in disease-free survival (P=0.05), but not in OS (P=0.23) following R0 resection (9).

The only factor which has been found to significantly correlate with OS is the clinical tumor stage (7). The application of adjuvant CTX in the present series was associated with lower OS than that following selective surgery alone (mean OS, 25.3, vs. 47.4 months).

By contrast, for palliative CTX, a beneficial effect has been demonstrated in various trials (10,12), particularly for fluoropyrimidine/oxaliplatin and modified FOLOFOX regimens (18,19). Furthermore, in late 2011, FOLFOX and CAPOX were confirmed effective with tolerable toxicity for advanced SBA (20,21).

Palliative surgical and/or chemotherapeutical treatment was applied to 21.2% of patients, either to reduce tumor-related intestinal obstruction or to slow down progression. Overall prognosis was poor and palliative treated patients showed a mean OS of only 9.8 months.

In 2007, studies on neoadjuvant radiochemotherapy showed the therapy had a tendency to improve OS rates in patients undergoing R0 resection compared with those who received selective surgical treatment (five-year OS, 83 vs. 53%; P=0.07) (13). Only one of the current patients received neoadjuvant CTX by application of gemcitabine, but a combined radiation therapy was not performed.

With regard to targeted therapies, particularly anti-epidermal growth factor receptor drugs, current literature only provides case reports (22,23). In 2008, Tsang *et al* described that the use of bevacizumab combined with gemcitabine and oxaliplatin in a patient with advanced unresectable SBA was beneficial. This lead to disease stabilization with a survival time of at least one year following diagnosis, considering a mean survival time of only 8–9 months in advanced SBA treated with standard chemotherapeutical care (22). One of the patients of the present study received bevacizumab combined with FOLFIRI, followed by FOLFOX regimen, in a palliative setting leading to a survival rate of 24 months.

In 2010, Overman *et al* reported cetuximab to be a promising candidate in the future SBA-treatment (9). However, prospective trials covering this topic are required.

Concordant with the current literature, the present study found that the presence of peritoneal carcinosis, lymph node metastases and increased disease stage impaired patient outcome. Furthermore, even following adequate surgical treatment, including R0 resection and sufficient lymphadenectomy, recurrence remained high leading to low survival rates. Although a multimodal therapeutic approach has been confirmed as the gold standard in the majority of solid malignancies, its value in the treatment of SBA has not yet been defined. With the exception of palliative CTX in unresectable stages, adjuvant CTX showed no survival benefit in the current series. The mean OS was longer in patients who had undergone selective surgery compared with patients who had received additive CTX.

Since the current literature does not provide any recommendations on tumor marker determination in SBA patients our records are incomplete. Due to non-specific initial symptoms and irregular observations in tumor marker levels, screening improvements were not detected in the present study.

SBA is rare but presents malignancies with extremely poor prognosis and often delayed diagnosis due to non-specific clinical signs and a lack of sufficient screening methods. As surgery remains the mainstay of treatment, the most important factor with regard to survival is R0 resection based on early diagnosis followed by early performed surgery. The role of adjuvant CTX in SBA treatment remains a matter of debate since no significant survival benefit has yet been confirmed. Encouraging translational study is inevitable for further hypothesis generation and for the development of new therapeutic approaches.

The results of the present study highlighted the urgent requirement for international collaboration and cross-national data provision to gain sufficient information on this specific patient population. We therefore demand a multinational data pooling to make a first step towards comprehensive information gathering, enabling us to offer the best evidence-based medical care to patients suffering from primary SBA in the future.

## Figures and Tables

**Table I:** Clinicopathohistological and therapeutic data: Survivors vs. non-survivors.

Variables (n=33)	Survivors (n=16)	Non-survivors (n=17)
Median age (IQR)	63 (24–78)	63 (42–83)
Gender, n (%)		
Male	8 (50.0)	12 (70.6)
Female	8 (50.0)	5 (29.4)
Tumor site, n (%)		
Duodenum	9 (56.3)	11 (64.7)
Duodenojejunal junction	1 (6.3)	1 (5.9)
Jejunum	5 (31.3)	3 (17.6)
Ileum	0	1 (5.9)
NOS	1 (6.3)	1 (5.9)
TNM grading, n (%)		
Poor (G3)	4 (25.0)	8 (47.1)
Moderate (G2)	9 (56.3)	9 (52.9)
High (G1)	3 (18.8)	0
Carcinosis peritonei, n (%)	0	9 (60.0)
R-status, n (%)		
R0	14 (87.5)	9 (52.9)
R1	1 (6.3)	0
No R-status (palliative GE)	0	5 (29.4)
NOS	1 (6.3)	3 (17.6)
pT, n (%)		
pT1	1 (6.3)	0
pT2	0	0
pT3	11 (68.8)	1 (5.9)
pT4	3 (18.8)	10 (58.8)
No surgery	0	2 (11.8)
NOS	1 (6.3)	4 (23.5)
pN, n (%)		
pN0	8 (50.0)	5 (29.4)
pN1/2	5 (31.3)	9 (52.9)
NOS	3 (18.8)	3 (17.6)
Chemotherapy, n (%)		
Adjuvant CTX	8 (50.0)	4 (23.2)
Palliative CTX	0	5 (29.4)
None	8 (50.0)	8 (47.1)
Surgical treatment, n (%)		
Segmental resection	14 (87.5)	6 (35.3)
Whipple^a^	2 (12.5)	4 (23.5)
Palliative GE	0	5 (29.4)
No surgery	0	2 (11.8)

a Pylorus-preserving pancreatoduodenectomy.

NOS, not otherwise specified; GE, gastroenterostomy. IQR, interquartile range; CTX, chemotherapy; R-status, resection status; R0, complete surgical resection; R1, incomplete surgical resection.

**Table II:** Therapeutic procedures: Survivors vs. non-survivors.

Therapeutic procedures	Survivors (n=16), n (%)	Non-survivors (n=17), n (%)
Whipple^a^	1 (6.3)	3 (17.6)
Whipple^a^ + CTX	1 (6.3)	1 (5.9)
Segmental resection	7 (43.8)	3 (17.6)
Segmental resection + CTX	7 (43.8)	3 (17.6)
GE	0	2 (11.8)
GE + CTX	0	3 (17.6)
Palliative CTX	0	2 (11.6)

a Pylorus-preserving pancreatoduodenectomy.

CTX, chemotherapy; GE, gastroenterostomy.

**Table III:** Patient characteristics and outcome data with regard to treatment performed.

Characteristics (n=33)	Surgery (n=14)	Surgery + CTX (n=12)	Palliative therapy (n=7)
Age, years, n	67.5	57.3	59.7
Males, n (%)	7 (50.0)	8 (66.7)	5 (71.4)
pT3, n (%)	7 (50.0)	5 (41.7)	0
pT4, n (%)	4 (28.6)	6 (50.0)	3 (42.9)
pN >0, n (%)	5 (35.7)	5 (41.6)	4 (57.1)
R0, n (%)	13 (92.9)	10 (83.3)	0
Carcinosis, n (%)	1 (7.7)	3 (25)	5 (83.3)
Mean OS, months	47.4	25.3	9.8
Mean survival time, months	19.3	19.6	9.8

CTX, chemotherapy; R0, complete surgical resection; OS, overall survival.
